# Association of adiponectin and metabolic syndrome in adolescents: the caspian- III study

**DOI:** 10.1186/s40200-015-0220-8

**Published:** 2015-12-18

**Authors:** Gita Shafiee, Zeinab Ahadi, Mostafa Qorbani, Roya Kelishadi, Hassan Ziauddin, Bagher Larijani, Ramin Heshmat

**Affiliations:** Chronic Diseases Research Center, Endocrinology and Metabolism Population Sciences Institute, Tehran University of Medical Sciences, Tehran, Iran; Department of Community Medicine, Alborz University of Medical Sciences, Karaj, Iran; Pediatrics Department, Child Growth and Development Research Center, Research Institute for Primordial Prevention of Non-communicable Disease, Isfahan University of Medical Sciences, Isfahan, Iran; Institute of Education and training, Tehran, Iran; Endocrinology and Metabolism Research Center, Endocrinology and Metabolism Clinical Sciences Institute, Tehran University of Medical Sciences, Tehran, Iran

**Keywords:** Adiponectin, Metabolic syndrome, Cardiometabolic, Pediatric

## Abstract

**Background:**

This study aimed to investigate the associations between metabolic syndrome (Mets) and adiponectin concentrations in Iranian adolescents.

**Methods:**

This study was conducted as a sub-study of a national school- based surveillance program in 10–18 year- old students from 27 provinces in Iran. Plasma adiponectin was measured in 180 randomly selected participants. Metabolic syndrome (Mets) was defined based on the Adult Treatment Panel III (ATP- III) criteria modified for the pediatric age group. Multiple logistic regression analyses were used to evaluate the association between adiponectin and cardiometabolic risk factors.

**Results:**

The median of adiponectin concentrations was significantly lower among participants with Mets [2.95 μg/ml (interquartile range 2.72–3.30)] compared with subjects without Mets [4.55 μg/ml (interquartile range 3.02–5.75)]. Adiponectin showed significant negative association with higher number of Mets components (*P*- trend < 0.05). Significant correlations were observed between adiponectin concentrations and metabolic parameters, except blood pressure.

Significant inverse association existed between adiponectin levels and presence of Mets (OR, 0.21; 95%CI: 0.10–0.45; *p* < 0.001). In multivariate models, this association remained significant after adjustment for other risk factors (OR, 0.18; 95%CI: 0.07–0.47; *p* < 0.001.

**Conclusions:**

Adiponectinhas inverse association with cardiometabolic parameters in Iranian adolescents, and it is a determinant of Mets independent of other risk factors. These findings can be used in comparison with other ethnic groups. Further longitudinal studies are necessary to assess the clinical impact of such inverse association.

## Background

Metabolic syndrome (Mets) is a cluster of cardiometabolic risk factors including abdominal obesity, dyslipidemia, hyperglycemia, and hypertension [1]. A growing body of evidence has shown that Mets increases cardiovascular disease (CVD) and all- cause mortality by 1.5–2 folds [2].

Mets is not limited to adults and exists in the pediatric age group [3], with higher prevalence in the in the Middle East than in Western countries [4].The increasing prevalence of the Mets and associated morbidities represents an important health problem world wide [5].

A number of obesity- related peptide hormones have been identified to play a role in the pathogenesis of Mets [6]. Adipokines, as adiponectin, are secreted by adipose tissue, which are associated with energy regulation, as well as glucose and lipid metabolism. Adiponectin has anti- inflammatory and anti- atherogenic effects and regulates metabolic homeostasis and vasodilatation, then it increases insulin sensitivity [7, 8]. Studies suggest that cardiometabolic risk factors as well as obesity- related disorders are mediated through adipokines associated with obesity [9, 10].

Studies conducted in pediatric populations have documented that plasma adiponectin concentrations have an inverse correlation with body mass index (BMI), fasting insulin [11, 12], and seem to be associated with a range of Mets’ components [13]. These findings suggest an etiologic role for low adiponectin levels in the pathophysiology of Mets [14]. Moreover, some studies have suggested that the associations between adiponectin and vascular risk factors vary across race and ethnic groups. Thus, it is necessary to obtain more data in diverse populations.

The aim of our study is to evaluate the association between adiponectin levels and cardiomatabolic risk factors in a representative sample of Iranian children and adolescents.

## Methods

We have previously described the method of the main study in detail [15]. Here we present it in brief and focus on the current sub-study.

The study sample was obtained as part of the third survey of a national surveillance program entitled “Childhood and Adolescents of Surveillance and PreventIon of Adult Noncommunicabledisease’ (CASPIAN- III) study. Overall 5088 students, aged 10–18 years, were selected through multistage random cluster sampling from urban and rural areas of 27 provinces in Iran. After a complete explanation of the study objectives and protocols for the students and their parents, written informed consent and verbal assent were obtained from parents and students, respectively.

### Clinical and biochemical measurements

Trained research assistants conducted the examinations, including measurements of anthropometric indexes and blood pressure (BP) under standard protocols by using calibrated instruments [15].

Venous blood samples were drawn from all study participants after 12 h of overnight fasting and delivered to the laboratory on the day of blood collection. Fasting blood glucose (FBG), total cholesterol (TC), high-density lipoprotein cholesterol (HDL-C), and triglycerides (TG) were measured enzymatically by auto-analyzers. HDL-C was determined after dextran sulfate-magnesium chloride precipitation of non-HDL-C. According to the Friedewald equation, low-density lipoprotein cholesterol l (LDL-C) was calculated in serum samples with TG ≤400 mg/dl. For the current sub-study, adiponectin was measured with the enzyme- linked immunosorbent assay (ELISA) kit (Abcam, USA) in 180 randomly selected sera. Intra and inter-assay CVs were 10 and 12 % for adiponectin, respectively.

### Criteria for mets

Subjects were classified as having Mets if they had at least three of the following criteria according to Adult Treatment Panel III (ATP III) criteria modified for the pediatric age group.

TG concentration of 150 mg/dL or greater; HDL-C concentration of40 mg/dL or less; FBG concentration of 100 mg/dL or greater; abdominal obesity: if waist to height ratio more than 0.5; and either systolic or diastolic BP (SBP, DBP) greater than the 90^th^ percentile for age, sex, and height [16].

### Statistical analysis

Normal distribution of continuous variables was assessed using Kolmogorov- Smirnov test. Means ± standard deviation (SD) or median (interquartile range) was used to express standard descriptive statistics. Categorical variables are expressed as percentages.

Differences between groups were investigated by T-test, and Mann- Whitney test in continuous variables with normal distribution and without normal distribution, respectively. Percentages of the categorized variables were compared using the Pearson Chi-square test. Spearman correlation was applied to determine the correlation between adiponectin and cardiometabolic parameters. Logistic regression analyses were used to evaluate the association between the Mets and adiponectin concentrations and other cardio-metabolic risk factors in each model by adjustment for possible confounders. All statistical analyses were performed by the SPSS statistical package for Windows (version 16.0, SPSSInc., Chicago, Illinois). *P* ≤ 0.05 was considered as statistically significant.

## Results

The study comprised 180 individuals (44.4 % boys) with mean ± SD age of 14.32 ± 2.6 years. The median of adiponectin concentrations was 2.95 μg/ml (interquartile range 2.72–3.30) and 4.55 μg/ml (interquartile range 3.02–5.75) in those with and without Mets, respectively. Table 1 presents the anthropometric and cardiometabolic characteristics of the participants. Fifteen percent of girls and 21.2 % of boys had Mets. Other than SBP and DBP, the mean ± SD of cardiometabolic factors was higher in those with Mets than those without the Mets. Participants with Mets had also significantly lower serum levels of adiponectin (*P* < 0.001).

**Table 1:** Baseline anthropometric and metabolic parameters of studied groups

	Mets – (*n* = 148)	Mets + (*n* = 32)	*P* - value
Age (Yr)	14.19 ± 2.64	14.91 ± 2.32	0.16
Weight (Kg)	42.41 ± 16.53	55.06 ± 17.81	<0.001
Height (Cm)	151.07 ± 14.46	156.94 ± 11.49	0.03
BMI (Kg/m^2^)	17.93 ± 4.28	21.85 ± 4.86	<0.001
WC (cm)	64.51 ± 9.20	75.50 ± 13.71	<0.001
Waist- to -height ratio	0.43 ± 0.04	0.48 ± 0.07	<0.001
SBP (mmHg)	99.68 ± 12.87	105.23 ± 19.49	0.07
DBP (mmHg)	63.91 ± 9.83	64.54 ± 11.62	0.77
FPG (mg/dL)	93.47 ± 9.69	108.75 ± 5.51	0.16
TG (mg/dL)	91.61 ± 37.26	143.10 ± 65.26	<0.001
HDL-C (mg/dL)	66.53 ± 20.78	44.06 ± 13.78	<0.001
LDL-C (mg/dL)	71.39 ± 32.37	96.90 ± 26.17	<0.001
Total Cholesterol (mg/dL)	148.20 ± 37.54	170.75 ± 26.58	0.002
Adiponectin (μg/ml)^a^	4.55(3.02–5.75)	2.95(2.75–3.30)	<0.001

Values are presented as mean ± standard deviation

^a^Value is median with interquartile rung

*Mets* metabolic syndrome, *BMI* body mass index, *WC* waist circumference, *SBP* systolic blood pressure, *DBP* diastolic blood pressure, *HDL-C* high-density lipoprotein cholesterol, *TG* triglycerides, *FBG* fasting blood glucose, *LDL-C* low density lipoprotein cholesterol

The correlation between adiponectin concentrations and metabolic parameters are shown in Table 2. A significant negative correlation existed between adiponectin and BMI, waist circumference (WC), abdominal obesity, TG, total cholesterol, and relatively strong correlation with FBG (*r* = −0.72, *P* < 0.001), LDL-C (*r* = −0.55, *P* < 0.001), and HDL-C (*r* = +0.65, *P* < 0.001).

**Table 2:** Spearman correlation of adiponectin levels with cardiometabolic parameters

	Boys		Girls		Total	
	r	*P* value	r	*P* value	r	*P* value
BMI (kg/m^2^_)_	−0.32	0.004	−0.12	0.24	−0.21	0.004
WC (cm)	−0.23	0.04	−0.16	0.12	−0.19	0.009
Abdominal obesity	−0.13	0.27	−0.25	0.01	−0.18	0.02
SBP (mmHg)	−0.03	0.82	−0.15	0.15	−0.10	0.20
DBP (mmHg)	−0.11	0.34	−0.15	0.15	−0.05	0.54
HDL-C (mg/dl)	+0.67	<0.001	+0.64	<0.001	+0.65	<0.001
TG (mg/dl)	−0.19	0.10	−0.13	0.18	−0.15	0.05
FBG (mg/dl)	−0.73	<0.001	−0.71	<0.001	−0.72	<0.001
LDL-C (mg/dl)	−0.52	<0.001	−0.58	<0.001	−0.55	<0.001
Total cholesterol (mg/dl)	−0.37	0.001	−0.30	0.002	−0.32	<0.001

*BMI* body mass index, *WC* waist circumference, *SBP* systolic blood pressure, *DBP* diastolic blood pressure, *HDL-C* high-density lipoprotein cholesterol, *TG* triglycerides, *FBG* fasting blood glucose, *LDL-C* low density lipoprotein cholesterol

Moreover, significant correlations were observed among both genders regarding HDL-C, FBG, LDL-C, and total cholesterol. Adiponectin significantly associated with BMI and WC in boys, but not in girls.

**Fig. 1:**
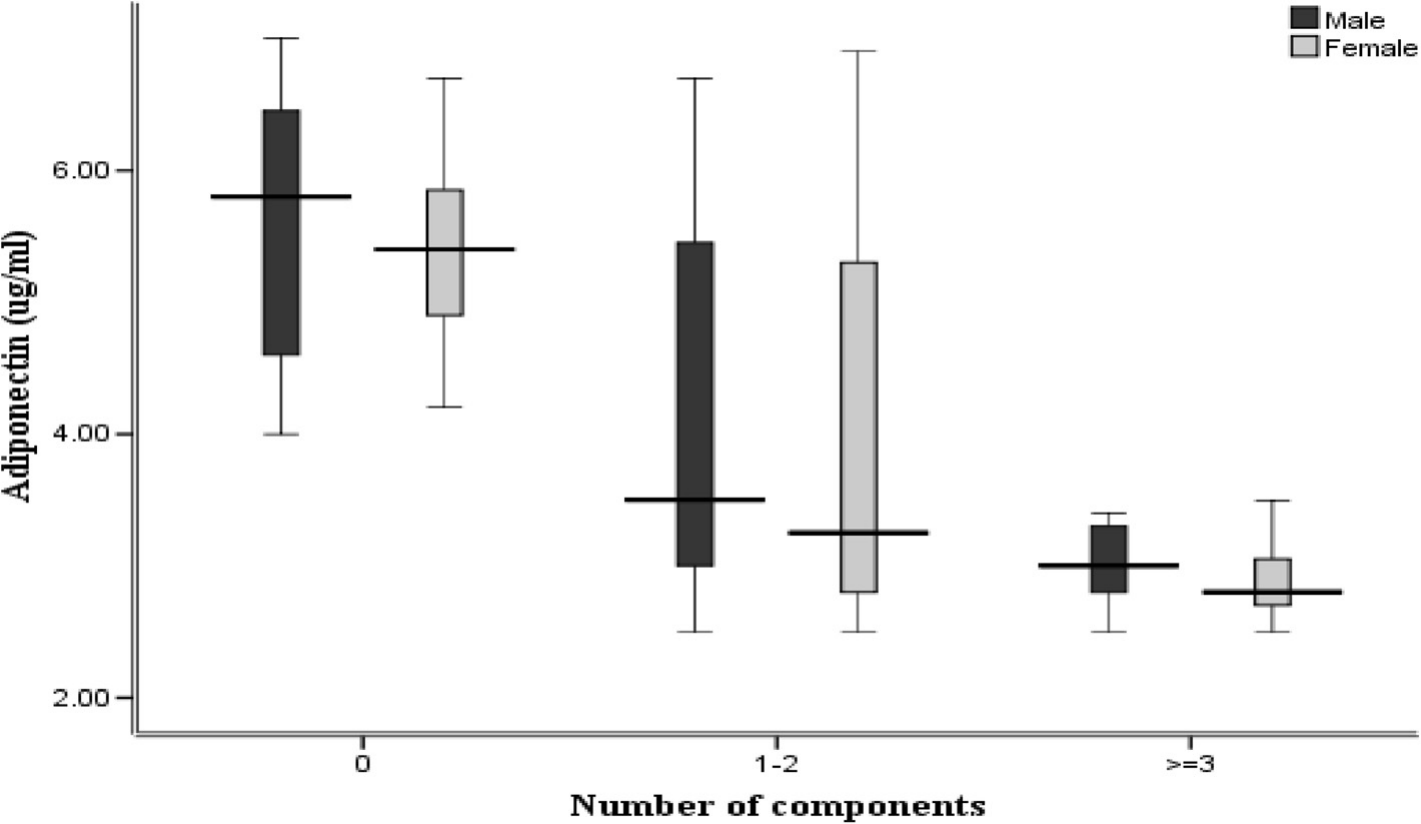
Adiponectin concentrations according to the number of metabolic syndrome components in girls and boys

As depicted in Fig. 1, a decreasing trend was observed in adiponectin concentrations according to the number of abnormal metabolic factors from 0–1–2, and to ≥3 in both boys and girls (*P* for trend <0.001).

Logistic regression models investigating the association between adiponectin and Mets are presented in Table 3. A significant inverse associationwas documented between adiponectin levels and the presence of Mets (OR, 0.21; 95%CI: 0.09–0.45; *p* < 0.001). This association remained significant after adjusting for other risk factors. In an additive multivariate model, after adjustment for age, BMI, total cholesterol and WC, this association remained significant between adiponectin and Mets in boys (OR, 0.14; 95%CI: 0.03–0.68; *P* = 0.01) and in girls (OR, 0.15; 95%CI: 0.03–0.64; *P* = 0.01).

**Table 3:** Association of serum adiponectin concentrations with metabolic syndrome in Iranian adolescents

	Boys		Girls		Total	
	OR (95%CI)	*P*- value	OR (95%CI)	*P*- value	OR (95%CI)	*P*- value
Model 1	0.19(0.06–0.59)	0.004	0.18(0.05–0.64)	0.008	0.21(0.10–0.45)	<0.001
Model 2	0.19(0.06–0.61)	0.005	0.16(0.05–0.59)	0.006	0.20(0.09–0.43)	<0.001
Model 3	0.15(0.04–0.66)	0.012	0.16(0.04–0.64)	0.009	0.19(0.08–0.46)	<0.001
Model 4	0.15(0.03–0.68)	0.014	0.18(0.05–0.72)	0.015	0.21(0.08–0.51)	0.001
Model 5	0.14(0.03–0.68)	0.014	0.15(0.03–0.64)	0.01	0.18(0.07–0.47)	<0.001

*OR* odds ratio, *CI* confidence interval

Model 1: crude model

Model 2: adjustment for age

Model 3: adjustment for age, body mass index (BMI)

Model 4: adjustment for age, BMI, total cholesterol

Model 5: adjustment for age, BMI, total cholesterol and waist circumference

## Discussion

The current study documented an inverse and independent association of adiponectin with cardiometabolic factors in adolescent girls and boys. Our findings are in line with previous studies that had shown a significant association between lower adiponectin concentrations with the risk of Mets development in healthy and type 2 diabetic adults [17, 18].

A previous study among adults reported [19] a negative association between adiponectin and BMI, WC, FBG and TG. It was also positively associated with HDL-C. Adiponectin has insulin- sensitizing effects through increasing 5’–AMP-activated protein kinase (AMPK) activation in the peripheral tissues [20]. These effects stimulate fatty acid oxidation in muscle cells and suppress glucose production in liver [21]. Therefore, adiponectin is one of the insulin- sensitizing hormones strongly associated with insulin resistance- related disorders including Mets [22]. Moreover, our findings on the association of adiponectin with lipid profile are in accordance with a growing body of literature suggesting that adiponectin has a direct effect on the regulation of lipid metabolism through its effect on lipoprotein lipase activity [23, 24]. Two independent studies showed that circulating adiponectin concentrations are negatively correlated with TG and positively correlated with HDL-C [24, 25]. Therefore, it might be of particular importance to consider adiponectin levels in primary prevention of atherosclerotic events associated with Mets [22].

Although we did not document any significant relation of adiponectin levels with SBP and DBP, several studies in adult populations reported inverse correlation of adiponectin with hypertension in healthy or diabetic individuals [26, 27].

Our finding on the inverse association of adiponectin with WC and abdominal obesity is consistent with some previous studies among adults [28]. It seems that the influence of adiponectin concentrations in intra-abdominal fat mass is greater than its influence on subcutaneous fat. As suggested by a previous experimental study, adiponectin administration induces weight loss, such effect has been independent of reducing the food intake in mice fed with high- fat diet [22]. The significant inverse association of adiponectin level with WC, BMI, and abdominal obesity in our study might be explained by the independent effect of adiponectin on weight loss [22].

We further investigated the adiponectin- Mets association by controlling it for other cardiometabolic parameters, as confounding factors. On all models, adiponectin increased the risk of Mets independent of age, BMI, WC, and total cholesterol. Our results support the findings of other studies that have analyzed the relationship between adiponectin concentrations and Mets [29, 30]. This association might be the effect ofadiponectinon Mets components. Adiponectin promotes to decrease TG levels, increasing glucose uptake by skeletal muscle and increasing HDL-C by an effect on hepatic lipase activity [31].

Ethnic differences are major factors in determining the expression level of adiponectin and its association with metabolic disorders as Mets [32, 33]. Various genetic backgrounds in different ethnicity may reflect on the relation between adiponectin and various cardiometabolic risk factors [34, 35]. The available data regarding the adiponectin concentrations in an Iranian adolescent population are scarce. Therefore, this study represents an important landmark in relating to adiponectin with Mets in Iran.

The present study has a number of limitations. It was cross-sectional and we could not explore a causal relation between adiponectin concentrations and development of Mets in adolescents. Another limitation of our study is that we did not consider dietary factors which may play important roles in the association adiponectin and Mets. The strengths of our study are its novelty in the Middle Eastern population and studying the adolescent age group.

## Conclusion

The study highlights the inverse and independent association ofadiponectin with the presence of Mets in Iranian adolescents. The major finding of our study was thatadiponectinexhibited a significantly higher probability of having Mets and was independent of BMI and other factors. Some physicians may have problems with including another criterion for the definition of the metabolic syndrome. In conclusion, we suggest that adiponectin would be a useful marker for identification the Mets.

These findings can be used in comparison with other ethnic groups. Further longitudinal studies are necessary to assess the clinical impact of such inverse association.

## References

[1] Reaven GM (2011). The metabolic syndrome: time to get off the merry-go-round?. J Intern Med.

[2] Mottillo S, Filion KB, Genest J, Joseph L, Pilote L, Poirier P (2010). The metabolic syndrome and cardiovascular risk a systematic review and meta-analysis. J Am Coll Cardiol.

[3] Johnson WD, Kroon JJ, Greenway FL, Bouchard C, Ryan D, Katzmarzyk PT (2009). Prevalence of risk factors for metabolic syndrome in adolescents: National Health and Nutrition Examination Survey (NHANES), 2001–2006. Arch Pediatr Adolesc Med.

[4] Delavari A, Forouzanfar MH, Alikhani S, Sharifian A, Kelishadi R (2009). First nationwide study of the prevalence of the metabolic syndrome and optimal cutoff points of waist circumference in the Middle East: the national survey of risk factors for noncommunicable diseases of Iran. Diabetes Care.

[5] Eckel RH, Grundy SM, Zimmet PZ (2005). The metabolic syndrome. Lancet.

[6] Matsuzawa Y, Funahashi T, Nakamura T (1999). Molecular mechanism of metabolic syndrome X: contribution of adipocytokines adipocyte-derived bioactive substances. Ann N Y Acad Sci.

[7] Tilg H, Moschen AR (2006). Adipocytokines: mediators linking adipose tissue, inflammation and immunity. Nat Rev Immunol.

[8] Wang ZV, Scherer PE (2008). Adiponectin, cardiovascular function, and hypertension. Hypertension.

[9] Gilardini L, McTernan PG, Girola A, da Silva NF, Alberti L, Kumar S (2006). Adiponectin is a candidate marker of metabolic syndrome in obese children and adolescents. Atherosclerosis.

[10] Chu NF, Wang DJ, Shieh SM, Rimm EB (2000). Plasma leptin concentrations and obesity in relation to insulin resistance syndrome components among school children in Taiwan--The Taipei Children Heart Study. International journal of obesity and related metabolic disorders : journal of the International Association for the Study of Obesity.

[11] Chu NF, Shen MH, Wu DM, Lai CJ (2005). Relationship between plasma adiponectin levels and metabolic risk profiles in Taiwanese children. Obes Res.

[12] Asayama K, Hayashibe H, Dobashi K, Uchida N, Nakane T, Kodera K (2003). Decrease in serum adiponectin level due to obesity and visceral fat accumulation in children. Obes Res.

[13] Valle M, Martos R, Gascon F, Canete R, Zafra MA, Morales R (2005). Low-grade systemic inflammation, hypoadiponectinemia and a high concentration of leptin are present in very young obese children, and correlate with metabolic syndrome. Diabetes Metab.

[14] Lara-Castro C, Fu Y, Chung BH, Garvey WT (2007). Adiponectin and the metabolic syndrome: mechanisms mediating risk for metabolic and cardiovascular disease. Curr Opin Lipidol.

[15] Kelishadi R, Heshmat R, Motlagh ME, Majdzadeh R, Keramatian K, Qorbani M (2012). Methodology and Early Findings of the Third Survey of CASPIAN Study: A National School-based Surveillance of Students’ High Risk Behaviors. International journal of preventive medicine.

[16] Zimmet P, Alberti G, Kaufman F, Tajima N, Silink M, Arslanian S (2007). The metabolic syndrome in children and adolescents. Lancet.

[17] Santaniemi M, Kesaniemi YA, Ukkola O (2006). Low plasma adiponectin concentration is an indicator of the metabolic syndrome. European journal of endocrinology / European Federation of Endocrine Societies.

[18] Yatagai T, Nagasaka S, Taniguchi A, Fukushima M, Nakamura T, Kuroe A (2003). Hypoadiponectinemia is associated with visceral fat accumulation and insulin resistance in Japanese men with type 2 diabetes mellitus. Metabolism: clinical and experimental.

[19] Abu-Farha M, Behbehani K, Elkum N (2014). Comprehensive analysis of circulating adipokines and hsCRP association with cardiovascular disease risk factors and metabolic syndrome in Arabs. Cardiovasc Diabetol.

[20] Kadowaki T, Yamauchi T (2005). Adiponectin and adiponectin receptors. Endocr Rev.

[21] Fang X, Sweeney G (2006). Mechanisms regulating energy metabolism by adiponectin in obesity and diabetes. Biochem Soc Trans.

[22] Blaslov K, Bulum T, Zibar K, Duvnjak L (2013). Relationship between Adiponectin Level, Insulin Sensitivity, and Metabolic Syndrome in Type 1 Diabetic Patients. International journal of endocrinology.

[23] Kazumi T, Kawaguchi A, Sakai K, Hirano T, Yoshino G (2002). Young men with high-normal blood pressure have lower serum adiponectin, smaller LDL size, and higher elevated heart rate than those with optimal blood pressure. Diabetes Care.

[24] Cnop M, Havel PJ, Utzschneider KM, Carr DB, Sinha MK, Boyko EJ (2003). Relationship of adiponectin to body fat distribution, insulin sensitivity and plasma lipoproteins: evidence for independent roles of age and sex. Diabetologia.

[25] Tschritter O, Fritsche A, Thamer C, Haap M, Shirkavand F, Rahe S (2003). Plasma adiponectin concentrations predict insulin sensitivity of both glucose and lipid metabolism. Diabetes.

[26] Baden MY, Yamada Y, Takahi Y, Obata Y, Saisho K, Tamba S (2013). Association of adiponectin with blood pressure in healthy people. Clin Endocrinol (Oxf).

[27] Shatat IF, Freeman KD, Vuguin PM, Dimartino-Nardi JR, Flynn JT (2009). Relationship between adiponectin and ambulatory blood pressure in obese adolescents. Pediatr Res.

[28] Isomaa B, Almgren P, Tuomi T, Forsen B, Lahti K, Nissen M (2001). Cardiovascular morbidity and mortality associated with the metabolic syndrome. Diabetes Care.

[29] Vega GL, Grundy SM (2013). Metabolic risk susceptibility in men is partially related to adiponectin/leptin ratio. Journal of obesity.

[30] Mente A, Meyre D, Lanktree MB, Heydarpour M, Davis AD, Miller R (2013). Causal relationship between adiponectin and metabolic traits: a Mendelian randomization study in a multiethnic population. PLoS One.

[31] Fuentes E, Fuentes F, Vilahur G, Badimon L, Palomo I (2013). Mechanisms of chronic state of inflammation as mediators that link obese adipose tissue and metabolic syndrome. Mediators Inflamm.

[32] Khan UI, Wang D, Sowers MR, Mancuso P, Everson-Rose SA, Scherer PE (2012). Race-ethnic differences in adipokine levels: the Study of Women’s Health Across the Nation (SWAN). Metabolism: clinical and experimental.

[33] Morimoto Y, Conroy SM, Ollberding NJ, Kim Y, Lim U, Cooney RV (2014). Ethnic differences in serum adipokine and C-reactive protein levels: the multiethnic cohort. Int J Obes (Lond).

[34] Saddi-Rosa P, Oliveira C, Crispim F, Giuffrida FM, de Lima V, Vieira J (2013). Association of circulating levels of nicotinamide phosphoribosyltransferase (NAMPT/Visfatin) and of a frequent polymorphism in the promoter of the NAMPT gene with coronary artery disease in diabetic and non-diabetic subjects. Cardiovasc Diabetol.

[35] Zadjali F, Al-Yahyaee S, Hassan MO, Albarwani S, Bayoumi RA (2013). Association of adiponectin promoter variants with traits and clusters of metabolic syndrome in Arabs: family-based study. Gene.

